# TRPV1 Contributes to the Neuroprotective Effect of Dexmedetomidine in Pilocarpine-Induced Status Epilepticus Juvenile Rats

**DOI:** 10.1155/2020/7623635

**Published:** 2020-04-07

**Authors:** Xingqin Tan, Yong Zeng, Zhenzhen Tu, Pan Li, Hengsheng Chen, Li Cheng, Shengfen Tu, Li Jiang

**Affiliations:** ^1^Pediatric Research Institute; Ministry of Education Key Laboratory of Child Development and Disorders, National Clinical Research Center for Child Health and Disorders (Chongqing), China International Science and Technology Cooperation Base of Child Development and Critical Disorders, Chongqing Key Laboratory of Pediatrics, Children's Hospital of Chongqing Medical University, Chongqing 400014, China; ^2^Department of Anesthesiology, Children's Hospital of Chongqing Medical University, Chongqing 400014, China; ^3^Emergency Department, The 2nd Affiliated Hospital of Chongqing Medical University, Chongqing 400014, China; ^4^Department of Neurology, Children's Hospital of Chongqing Medical University, Chongqing 400014, China

## Abstract

To investigate the antiepileptic and neuroprotective effects of dexmedetomidine (Dex) in pilocarpine- (Pilo-) induced status epilepticus (SE) juvenile rats, rats were randomly assigned to the following six groups (*n* = 20): normal, normal+Dex, SE, SE+Cap, SE+Dex, and SE+Dex+Cap. The rats were treated with either diazepam (i.p., an antiepileptic drug) or Dex after the onset of SE. The Morris water maze was used to assess rat cognitive behavior. Flow cytometry was used to detect the concentrations of Ca^2+^, mitochondrial membrane potential, and reactive oxygen species. Transmission electron microscopy was performed to evaluate specimens of brain tissue. The levels of caspase 3 and TRPV1 were examined by western blot and immunohistochemistry (IHC). Treatment with Dex significantly decreased the escape latency of the SE rats (*P* < 0.05). Capsaicin, a TRPV1 agonist, delivery aggravated the performance of SE rats. Pathological changes in SE rat were attenuated by Dex and deteriorated by capsaicin. Swollen mitochondria and abnormal endoplasmic reticulum were found in SE rats and were then aggravated by capsaicin and reversed by Dex. Moreover, our data showed that Dex significantly restrained calcium overload, ROS production, and mitochondrial membrane potential loss, all of which were induced by Pilo and capsaicin (*P* < 0.05). Dex decreased the apoptotic rate in the Model SE group (*P* < 0.05) and TRPV1 and caspase 3 expression in the Dex treatment group (*P* < 0.05). Interestingly, all these effects of Dex were partially counteracted by the TRPV1 agonist, capsaicin (*P* < 0.05). Our study showed that Dex exerted a neuroprotective effect in Pilo-induced SE rats by inhibiting TRPV1 expression and provided information for therapy to SE patients.

## 1. Introduction

Status epilepticus is the most common neurological emergency in children. It can cause irreversible brain damage due to excitotoxic damage of neurons and left behind sequelae such as epilepsy and cognitive impairment [1]. Patients with status epilepticus (SE) have a higher mortality rate (21-33%) than patients with generalized epilepsy, with death occurring within 30 days of the initial convulsant activity [2]. The incidence of SE in children was as high as ~20 per 100,000 individuals [3]. The existing antiepileptic drugs effectively control seizures by reducing neuronal excitability, but secondary brain damage including SE still exists in cognitive impairment [4]. In children, SE can cause neuronal cell loss, interfere with developmental progress, lead to epilepsy and cognitive impairments, and seriously influence the child's quality of life [4]. In the immature brains of children, frequent or prolonged seizures often result in irreversible brain damage and long-lasting sequelae [4]. Therefore, it is necessary to perform brain protection treatment to injury induced by SE in children.

Transient receptor potential (TRP) channels are expressed in a specific brain region, such as the hippocampus and hypothalamus [5, 6]. Studies reported that TRPV1 receptors are involved in the etiopathogenesis of epilepsy [7–10]. TRPV1 receptors are overexpressed in the dentate gyrus of pilocarpine-induced SE mice [11] and also in the temporal cortex and hippocampal tissues of patients with mesial temporal lobe epilepsy [12]. Either activation of VGCC (calcium channel, voltage-dependent, beta 3 subunit) and NMDA receptors or cytoplasmic Ca^2+^ is also elevated, resulting in a large amount of Ca^2+^ introduced through the TRP channel, including the TRPV1 channel, and triggering the biochemical cascade and leading to acute neuronal cell death as a result [13]. Studies also concluded that TRPV1 can be a potential target in the treatment of epilepsy [14, 15].

Dexmedetomidine is a highly selective central agonist of *α*2-adrenergic receptors, as well as an anxiolytic, sedative, and analgesic medication that produces beneficial effects on respiration and neuroprotection [16]. Zhai et al. have found that high-dose Dex can reduce the number and cumulative time of seizures in rats with epileptic seizures [17]. At the same time, it can alleviate glutamate in the hippocampus, upregulate oxidized glutathione, and finally show anticonvulsant and brain protection characteristics [18]. Dex can inhibit the activation of TRPV1 and TRPM2 (transient receptor potential cation channel subfamily M member 2) channels, reduce the calcium influx caused by oxidative stress damage, inhibit the generation of oxygen free radicals and mitochondrial apoptosis, and achieve brain protection in animal models of cerebral ischemia [19]. Our previous study has demonstrated that Dex can attenuate excitatory nerve damage caused by SE, reduce high metabolic level of SE, and exert antioxidative stress function [20].

In this study, the neuroprotective effect of Dex in a SE rat model was investigated. Pilo was used to induce the SE rat model, and a TRPV1 agonist, capsaicin, was involved in SE rats. This research will illustrate that TRPV1 might be a potential target for treating SE and also provide the theoretical basis in the therapeutic strategy of SE.

## 2. Materials and Methods

### 2.1. Animals

All animal procedures were carried out in accordance with the National Institutes of Health guidelines and were approved by the Ethics Committee of Chongqing Medical University Animal Center (Chongqing, China). A total of 100 healthy, male, immature Sprague Dawley rats (age, 20 days; weight, 42-52 g; animal certificate No. SCXK (Yu) 2012-0001; specific pathogen free (SPF)) were provided by Chongqing Medical University Animal Center and housed in a controlled environment (food and water available *ad libitum*, 21 ± 1°C, 60% humidity, lights on from 7:00 AM–7:00 PM).

### 2.2. Preparation of the SE Model Rats and Sample Collection

**Figure 1:**
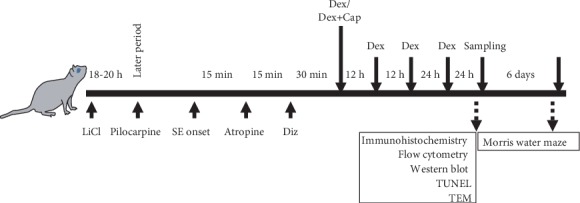
Outline of the experimental design in this research. Dex was delivered at 1 h, 12 h, 24 h, and 48 h after SE onset.

The rats were randomly assigned to 6 different groups (*n* = 20 per group): normal (healthy rats administered saline alone), normal+Dex (healthy rats treated with 0.2 *μ*g/g Dex), SE model (rats treated with 30 mg/kg Pilo), SE+Cap (SE model rats treated with capsaicin), SE+Dex (SE rats treated with Dex), and SE+Dex+Cap (SE rats treated with capsaicin and Dex). The SE model was established by the lithium chloride-pilocarpine method, as previously described [21]. Rats in all the groups except the normal group received either 10 mg/kg diazepam (Tianjin Jinyao Amino Acid Co., Ltd., Tianjin, China) or Dex by intraperitoneal injection 60 min following the onset of SE. Capsaicin at a dose of 0.1 *μ*g/g was also delivered in certain groups. Dex was delivered, at 12 h, 24 h, and 48 h after the first injection of Dex. Diazepam (10 mg/kg, i.p.) was used to stop the convulsions that occurred after SE induction. SD rats that had not experienced Class 4 seizures by 30 min after the intraperitoneal injection of pilocarpine were treated with a second dose of 10 mg/kg pilocarpine; the rats that failed to experience seizures after the second dose were excluded from the study. In addition, control rats were intraperitoneally injected with lithium, atropine, or chloral hydrate, without pilocarpine. Atropine was delivered at a dose of 1 mg/kg to reduce peripheral effects according to the administered strategy shown in Figure 1. For further transmission electron microscopy, flow cytometry, western blotting immunohistochemistry, and Terminal-deoxynucleoitidyl Transferase-Mediated Nick End Labeling (TUNEL) staining were collected 24 h after the latest drugs intraperitoneally. An animal ethology test, including Morris water maze, was started one week after the latest drugs intraperitoneally. The outline of the experimental design in this research is also shown in Figure 1.

### 2.3. Morris Water Maze

Morris water maze (*n* = 6) studies were performed as previously described by Han et al. [21]. To measure the acquisition of learned behavior, navigation trials were performed on a daily basis (5 trials/day) with each rat for 5 consecutive days. During the training period, the rats were trained to swim to a hidden platform that was situated below the surface of the water and located at the center of one of the four quadrants. During the four trials, each rat was randomly placed in one of the four starting positions and then given 1 min to find the platform and stay on it for 10 sec. If the animal failed to find the platform within the given time period, it was gently guided to the platform and allowed to stay on it for 10 sec. Each rat was trained to find the hidden platform in the pool during 10 trials. To evaluate the rat's spatial retention ability, space probe trials were carried out on the 6th day. The platform was removed, and the total distance traveled while swimming and the distance swam in the target quadrant for 1 min were recorded by a tracking system.

### 2.4. Transmission Electron Microscopy

The rat whole brains were prepared and analyzed as previously described [22]. Briefly, samples of rat brain tissue were fixed in a solution containing 3% glutaraldehyde, 1% OsO_4_, and 1.5% K_4_Fe (CN)_6_ (potassium ferrocyanide-reduced osmium). The samples were then embedded with 1% agar gel and epoxy resin. Tissue sections across the hippocampus were cut with a diamond knife and stained with a solution containing Reynolds lead citrate and 1% uranyl acetate. Finally, the sections were observed by using a Morgagni 268 transmission electron microscope (FEI Company, Eindhoven, The Netherlands) at 80 kV. Three rats in each group were obtained for transmission electron microscopy detection.

### 2.5. ROS (Reactive Oxygen Species), Ca^2+^ and JC-1 Detection, and Flow Cytometry

Brain tissues were obtained from the rats in each group and cut into pieces in PBS solution under aseptic conditions. Collagenase and trypsin were added to achieve single-cell suspensions after sieving using 48 *μ*m nylon fine screen. For ROS detection, cells were incubated with 10 mM DCFH-DA (Beyotime Institute of Biotechnology) at a final concentration for 60 min at 37°C and then washed 2 times with a serum-free medium in the dark. For the determination of intracellular free calcium ([Ca^2+^]i), cells were loaded with fluo-4-AM (KeyGen Biotech, Jiangsu, China) at a concentration of 3 *μ*M and then incubated for 30 min at 37°C and then washed with Ca^2+^-free buffer; after which, they were resuspended at a final volume of 0.5 mL. The concentration of intracellular free Ca^2+^ was determined as described by Bolnick J. M [23]. For mitochondrial membrane potential (JC-1) analysis, the cells were incubated with 0.5 mL JC-1 working reagent (Yeasen Bio, Shanghai, China) at 37°C for 15 min; after which, the cells were centrifuged (400 g, 5 min), and the supernatant was discarded. Following centrifugation, the cell pellets were suspended in PBS for analysis by flow cytometry (BD Biosciences, San Jose, CA, USA). Three rats in each group were obtained for flow cytometry.

### 2.6. Western Blotting

Western blot assays were performed as previously described by Gong et al. [24]. The primary antibodies used for immunostaining were TRPV1 antibody (bs-23926R; 1 : 300 dilution, Bioss antibody, Beijing, China), caspase 3 antibody (bs-0081R; 1 : 300 dilution, Bioss antibody, Beijing, China), and GAPDH antibody (Boster, BA2913; 1 : 10000, Pleasanton, CA, USA). A secondary horseradish peroxidase-conjugated AffiniPure anti-rabbit antibody (ZSGB-Bio, Beijing, China). Detailed procedures for immunoblotting are described [19]. The optical density of western blot bands was quantified using Image-Pro Plus 6.0. Three rats in each group were obtained for western blot assay.

### 2.7. Immunohistochemistry

The staining protocol employed a modified streptavidin-HRP immunohistochemistry procedure (CoWin Century Biotechnology, Inc., Beijing, PRC). Briefly, slide-mounted 4 *μ*m thick tissue sections were incubated overnight with rabbit anti-rat caspase 3 polyclonal antibodies (bsm-33199 M; 1 : 400 dilution, Bioss antibody, Beijing, China); after which, they were incubated with peroxidase-conjugated streptavidin and then visualized by using reagents in a diaminobenzidine (DAB) staining kit (CoWin Century Biotechnology, Inc., Beijing, PRC). Three rats in each group were obtained for immunohistochemistry. Image-Pro Plus 6.0 software was used for the quantification of a positive area. The area of cells in claybank was regarded as the positive area.

### 2.8. Terminal-Deoxynucleoitidyl Transferase-Mediated Nick End Labeling Staining

TUNEL staining was performed according to instructions provided by the manufacturer of an in situ cell death detection kit (Roche Diagnostics, Indianapolis, IN, USA). Tissue sections were deparaffinized, rehydrated, and washed with PBS for 10 min. Next, the sections were washed with permeabilization solution (0.1% Triton X-100, 0.1% sodium citrate) for 5 min and then washed with PBS. Next, the sections were incubated with TUNEL reaction mixtures I and II for 10 min at 25°C in a humidified chamber; after which, they were washed with PBS and incubated for 30 min with converter-POD. The sections were then mounted on slides and stained with diaminobenzidine (DAB), followed by counterstaining with hematoxylin. Finally, the sections were examined under a light microscope (Nikon, Tokyo, Japan). Three rats in each group were obtained for TUNEL assay. The area of the cell nucleus in claybank was regarded as the positive area.

### 2.9. Statistical Analysis

All statistical analyses were performed using IBM SPSS Statistics for Windows, version 22 (IBM Corp., Armonk, NY, USA). Results are expressed as the mean ± standard deviation (SD). Comparisons between multiple groups were analyzed by the Kruskal-Wallis test. A *P* value < 0.05 was considered statistically significant. Six animals were included in an animal ethology test, and other experiments, such as western blot, TEM, flow cytometry, and IHC, in this study were repeated for three times at least.

## 3. Results

### 3.1. Dex Improved Memory and Cognition in SE Rats

**Figure 2:**
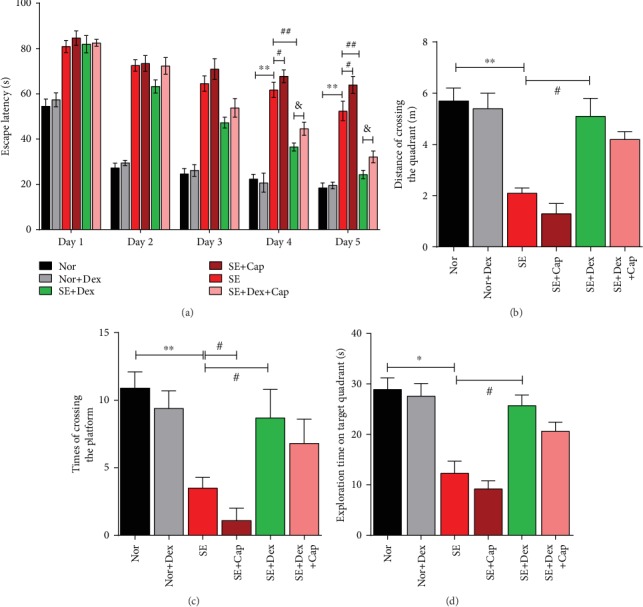
Dex improved memory and learning in Pilo-induced SE rats, while these effects were blocked by capsaicin. The Morris water maze was performed 9 days after SE onset. (a) The escape latency of the rats in each group and (b) the swimming distance for the rats in each group. (c) The times the rats took in crossing the platform in each group. (d) Exploration time of the rats in the target quadrant in each group. All data are reported as the mean ± SD (*N* = 6, ^∗^*P* < 0.05 and ^∗∗^*P* < 0.01 vs. the normal group; ^#^*P* < 0.05 and ^#^^#^*P* < 0.01 vs. the SE model group; ^&^*P* < 0.05 vs. the SE+Dex group).

Plots of escape latency in each group are shown in Figure 2(a) and indicated a gradual decrease in latency times after 5 days of training. Specifically, the mean escape latency in the SE model group was significantly longer than those in the normal and normal+Dex groups (*P* < 0.01) and further lengthened by capsaicin in SE rats. However, the mean escape latency in the SE+Dex group was significantly shorter than that in the SE model group (*P* < 0.01), which was reversed by capsaicin in SE+Dex rats (*P* < 0.05). Furthermore, on the 6th day, after removing the platform, rats with SE presented a significantly short distance compared with SE rats treated with Dex (Figure 2(b)) and which traveled more times crossing platforms (Figure 2(c)) and longer time in the target quadrant (Figure 2(d)) than rats in the SE group (*P* < 0.05). Time stay in the target quadrant of rats in SE+Cap was further less than that of SE model rats. However, all the beneficial effects of Dex on escape latency were blocked by treatment with the TRPV1 agonist, capsaicin (*P* < 0.05), while no significant difference was found between SE+Dex+Cap and SE+Dex on the distance of crossing, times of crossing the platform, and time stay in the target quadrant (*P* > 0.05).

### 3.2. Dex Alleviated the Histopathological Changes Observed in SE Rats

**Figure 3:**
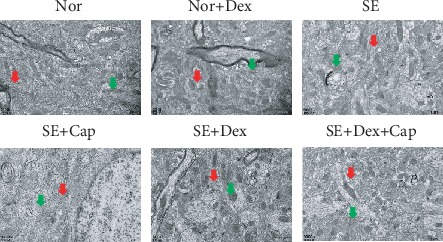
Dex alleviated the morphological changes in the hippocampal CA1 area of rats. Cellular ultrastructure of a rat brain was observed by transmission electron microscopy 72 h after SE onset. Red arrow: endoplasmic reticulum; green arrow: mitochondria. *N* = 3.

The ultrastructure of the brain has reported to present a change in SE rats compared with normal rats [25]. In this study, in both the normal and normal+Dex groups, the neurons contained large numbers of endoplasmic reticulum (red arrow) and mitochondria (green arrow), as well as substantial amounts of smooth surface endoplasmic reticulum, and the neuronal mitochondria displayed a homogeneous matrix (Figure 3). In addition, myelinated nerve fibers and large numbers of astrocytes and protruding neuropils were observed. In the model SE group, the mitochondria in the neurons were swollen, the coarse surfaces of the endoplasmic reticulum were dilated, myeloid tissue was degenerated, the nucleoli were dissolved, the nuclear membranes were ruptured, cytoplasmic edema was observed, and the cytoplasm in astrocytes was swollen and enlarged. Cells presented necrotic state after SE rats were treated with capsaicin, and capsaicin exerted a deteriorating effect compared with SE rats. Afterwards, Dex rescued the abnormality of the endoplasmic reticulum and mitochondria in SE rats, while further treatment with capsaicin deteriorated the organelle disorder.

### 3.3. Dex Attenuated Intracellular Ca^2+^, ROS Production, and Mediated JC-1 Levels in SE Rats

**Figure 4:**
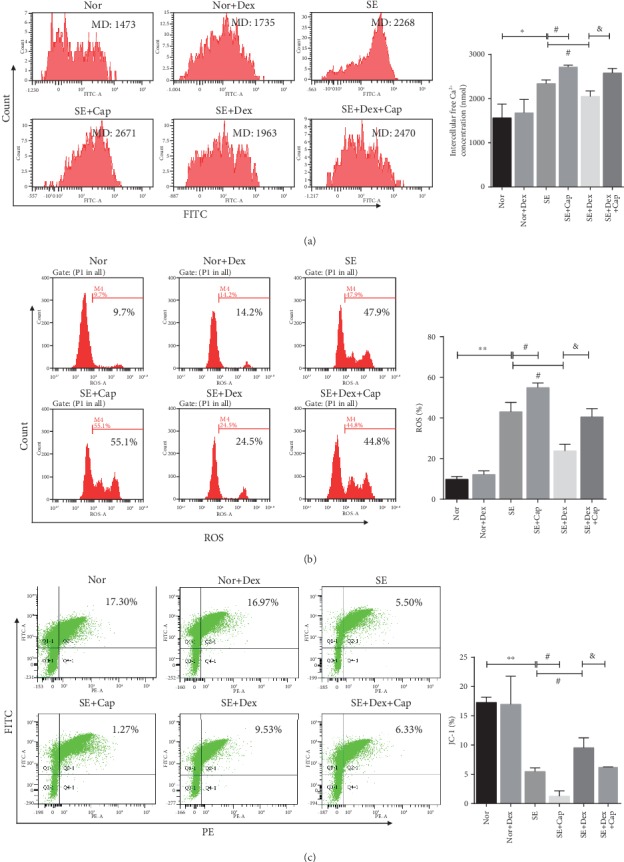
Effect of Dex on [Ca^2+^]i concentration, ROS production, and JC-1 depolarization in the brains of rats. (a) [Ca^2+^]i concentrations were measured by UV light-excitable Fura-4 AM. (b) ROS production was detected by flow cytometry. (c) Mitochondrial membrane potential was determined by JC-1 staining. ^∗^*P* < 0.05 and ^∗∗^*P* < 0.01 vs. the normal group; ^#^*P* < 0.05 vs. the SE model group; ^&^*P* < 0.05 vs. the SE+Dex group. *N* = 3.

Studies suggested that ROS, JC-1, and intracellular Ca^2+^ are key modulators in SE [26, 27]. As shown in Figures 4(a), the [Ca^2+^]i concentrations in the brains of SE rats were much higher than those in the brains of rats in the normal group and normal+Dex group (*P* < 0.01). Further, capsaicin extended the value of [Ca^2+^]i concentrations in SE+Cap. Interestingly, the mean [Ca^2+^]i concentration in the SE+Dex group was significantly lower than that in the SE model group (*P* < 0.01). Moreover, the mean [Ca^2+^]i concentration in the SE+Dex+Cap group was higher than that in the Dex treatment group (*P* < 0.05).

The effect of Dex on ROS levels in the rat brains is shown in Figure 4(b). ROS production was significantly increased in the SE rats, and capsaicin can further enhance the ROS production, which was suppressed by Dex delivery. As expected, there was a significant increase in ROS production after the rats were treated with Cap in the SE+DEX group (Figure 4(b)). The JC-1 ratios of red to green fluorescence among rats in the normal and normal+Dex groups were 1.01 ± 0.04 and 0.98 ± 0.03, respectively, which were significantly higher than those among rats in the SE model group (0.47 ± 0.11, *P* < 0.01 vs. the normal group). As a comparison, rats in SE+Cap presented a severe mitochondrial membrane potential loss than SE rats (*P* < 0.05). After Dex treatment, the red to green fluorescence ratio was 0.84 ± 0.09, which was significantly higher than that in the SE model group (*P* < 0.01 vs. the SE model group). However, that ratio decreased after treatment with Cap (0.69 ± 0.14) (*P* < 0.05, vs. the SE+Dex group) (Figure 4(c)).

### 3.4. Dex Treatment Reduced Cell Apoptosis by Decreasing Caspase 3 and TRPV1 Expression in SE Rats

**Figure 5:**
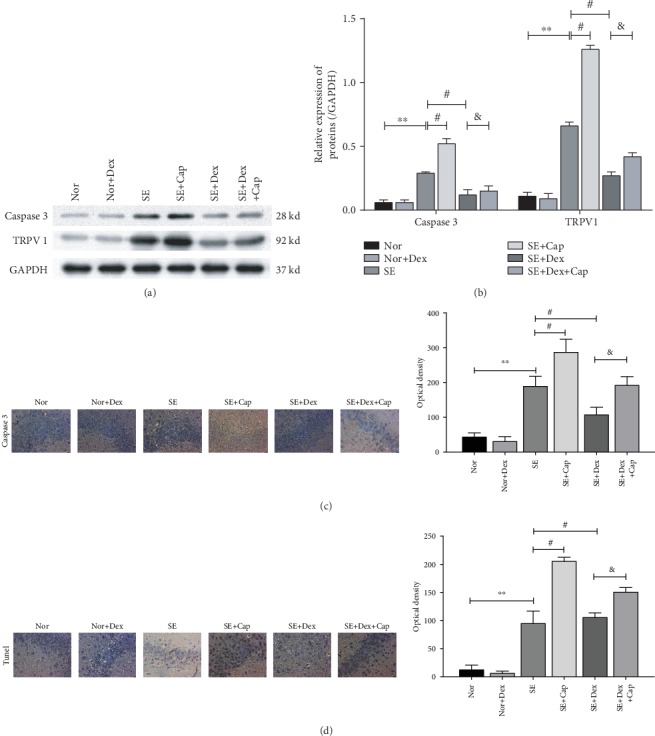
Dex treatment inhibited caspase 3 and TRPV1 expression and reduced cell apoptosis in the Pilo-induced SE rats. (a) Caspase 3 and TRPV1 expression was measured with western blot. (b) The expression levels of caspase 3 and TRPV1 protein. (c, d) Apoptosis was measured by detecting caspase 3 using IHC assay (c) and TUNEL staining (d) in the brains of the rats in each group; magnification, ×400. ^∗∗^*P* < 0.01 vs. the normal group; ^#^*P* < 0.05 vs. the SE model group; ^&^*P* < 0.05 vs. the SE+Dex group. *N* = 3.

To explore the expression of TRPV1 and caspase 3, the whole brain of rats of each group was obtained. As shown in Figure 5(a), the levels of caspase 3 and TRPV1 expression in the brain of rats in the SE group were significantly higher than those in the normal and normal+Dex groups.  Figure 5(b) represents data shown in Figure 5(a), while this expression was further increased by capsaicin in the SE group. After Dex treatment, the expression levels of both caspase 3 and TRPV1 decreased. However, treatment with Cap led to a remarkable reverse in caspase 3 and TRPV1 expression when compared to that in the SE+Dex group (Figures 5(a) and 5(b)). In addition, slides across the hippocampus were obtained for further IHC and TUNEL. As shown in Figure 5(c), there are more cells with caspase 3 expressed in the hippocampus of SE rats when compared to rats in the normal group and normal+Dex group. However, apoptotic cells in the SE group were increased by capsaicin administration and then decreased after Dex treatment (*P* < 0.05). Interestingly, capsaicin treatment reversed the inhibitive role of Dex on cell apoptosis (Figure 5(c)). Further, cell death was also detected using TUNEL staining. As displayed in Figure 5(d), apoptotic cells presented a similar trend shown in Figure 5(c).

## 4. Discussion

In this study, we found that the rat model of epilepticus induced by lithium chloride-pilocarpine showed that the latency of the platform was significantly prolonged in the water maze experiment, the exploration time in the quadrant of the original platform was reduced, the times of crossing platforms were reduced, and the swimming distance was shorter. This result is consistent with previous research results [28]. After the intervention of Dex, the latency of the model rats was shortened, the exploration time in the quadrant of the original platform was prolonged, the times of crossing platforms increased, and the swimming distance increased. It indicated that Dex improved the cognitive memory function of SE rats to some extent. In short, capsaicin, as a TRPV1 agonist, can reverse the protective role of Dex.

Dexmedetomidine participates in brain protection through antioxidation, inhibition of intracellular calcium overload, and mitochondrial protection. In the glutamate-induced PC12 cytotoxicity model, Dex pretreatment significantly reduced MDA content, enhanced SOD activity, inhibited ROS overproduction, decreased intracellular Ca^2+^ levels, and maintained stable mitochondrial membrane potential [28]. Interestingly, our results confirmed that oxidative stress, calcium overload and neuronal apoptosis were all evoked in the SE rats and were deteriorated by capsaicin treatment. Treatment with Dex blocked the influx of Ca^2+^, improved mitochondrial membrane potential, and inhibited the caspase 3 and TRPV1 expression, while afterwards protective functions were reversed by capsaicin administration. These results indicate that Dex exerts an antiapoptotic effect by mediating oxidative stress via regulation of ROS production and Ca^2+^ influx and improves mitochondrial membrane potential by mediating TRPV1.

Transient receptor potential vanilloid receptor I (TRPV1) is a member of the transient receptor potential family [14]. It is a nonselective cation channel with high calcium permeability that can be activated by noxious heat, pH changes, fatty acid amides, and endogenous lipid ligands [14]. Increasing evidence suggests that TRPV1 is involved in the regulation of synaptic plasticity, the synaptic remodeling of learning, memory process, and also many neurological diseases such as epilepsy, Alzheimer's disease, and Parkinson's disease [29]. A study has confirmed that epilepsy can induce hippocampal apoptosis, high expression of caspase 3 and caspase 9, ROS, mitochondrial depolarization, and increased intracellular calcium concentration. Administration of TRPV1 channel blocker capsazepine resulted in a decrease in intracellular calcium ion concentration. The administration of the TRPV1 channel agonist capsaicin increased intracellular calcium ions, leading to apoptosis in the rat dorsal root ganglion and hippocampus [30]. Actually, capsaicin, as an agonist of TRPV1, has been studied in a seizure model already. A new research by Naziroglu et al. indicates that intracellular free calcium ion is enhanced by capsaicin and cell apoptosis; expression of caspase 3 and 9 is also increased [8]. Besides, Carletti et al. also discovered that rats with temporal lobe epilepsy will have increased epileptic outcome after receiving a capsaicin administration [31]. A further study suggests that TRPV1 can be blocked by dexmedetomidine [19]. Besides, TRPV1 expression promotion might alter basal synaptic transmission in CA1 and CA3 of hippocampal alteration and thus induce field excitatory postsynaptic potentials (fEPSPs) ([15]), while some reports indicate that capsaicin has a potent antioxidative effect in vivo [32], and activation of TRPV1 by capsaicin can protect the kidney from damage by reducing inflammatory and oxidative stress [33]. This contrary conclusion might be due to the different roles of TRPV1. In this study, TRPV1 expression, together with caspase 3, was evoked in rats of SE, and capsaicin administration can enhance its expression further, while Dex delivery significantly inhibited TRPV1 and caspase 3 expression in the SE+Dex group and capsaicin reversed the protective role of Dex.

In conclusion, we discovered that TRPV1 was involved in Pilo-induced SE rats by inducing cell apoptosis, ROS production, mitochondrial membrane potential disorder, and intracellular Ca^2+^ overload. In addition, Dex attenuates memory impairment and neuronal degeneration in the brain. Its beneficial effect is partially due to its ability to mitigate oxidative stress by inhibiting TRPV1 activity and reversed by capsaicin treatment. While this study further confirmed the neuroprotective effect of Dex, additional studies are needed to elucidate the complete mechanism involved. The ability of Dex to inhibit Pilo-induced TRPV1 activation should be taken into consideration when searching for potential pharmacological targets for SE treatment. A further study will be taken into consideration in the role of TRPM2 and whether TRPV1 can be a direct target of Dex.

## Data Availability

The datasets used and/or analyzed during the current study are available from the corresponding author on reasonable request.

## Abbreviations

SE: Status epilepticus
Nor: Normal
Dex: Dexmedetomidine
Cap: Capsaicin
Diz: Diazepam
TEM: Transmission electron microscopy
TUNEL: Terminal-deoxynucleoitidyl Transferase-Mediated Nick End Labeling
TRPV1: Transient receptor potential cation channel subfamily V member 1
VGCC: Calcium channel, voltage-dependent, beta 3 subunit
TRPM2: Transient receptor potential cation channel subfamily M member 2.
